# Supplementary material for: Age at Onset and Presenting Symptoms of Neurofibromatosis Type 2 as Prognostic Factors for Clinical Course of Vestibular Schwannomas
Source: Cancers (Basel). 2020 Aug 20;12(9):2355. doi: 10.3390/cancers12092355 (PMC7563356; doi:10.3390/cancers12092355)
Supplement: Supplementary file 1 [file cancers-12-02355-s001.pdf]

Supplementary Materials

Age at Onset and Presenting Symptom of Neurofibromatosis Type 2 as Prognostic Factors for Clinical Course of Vestibular Schwannomas

Isabel Gugel, Florian Grimm, Julian Zipfel, Christian Teuber, Ulrike Ernemann, Lan Kluwe, Marcos Tatagiba, Victor-Felix Mautner and Martin Ulrich Schuhmann

Table S1. Parameters for the 106 NF2 patients.

	Patient No							
**Patient ID**	**1**	**2**	**3**	**5**	**7**	**8**	**9**	**11**
**Sex**	M	F	F	M	F	F	F	F
**Family history**	Negative	Negative	Negative	Negative	Positive (father)	Negative	Negative	Negative
***NF2* mutation type**	NA	FS	NS	FS	Large genome alteration	NS	NS	FS
**Age at signs of symptoms**	7	1	6	1	3	2	5	4
**Age at time of diagnosis**	8	10	6	1	3	3	7	13
**Age at start of treatment (L/R)**	13/9	13/17	11/14	11/13	10/11	14/14	11/12	14/19
**Age at time of surgery (L/R)**	13/9	13/17	11/14	11/13	10/11	14/14	11/12	14/16
**Age at deafness (L/R)**	-/-	-/-	-/-	19/-	-/-	16/-	-/-	14/25
**BVZ in the following course**	Yes	Yes	Yes	Yes	No	No	No	Yes
**Radiation in the following course (L/R)**	No/No	No/No	No/No	No/No	No/No	No/No	No/No	No/No
**Presenting symptoms**	Visual impairment, cutaneous plexiform schwannoma	Cutaneous plexiform schwannoma, seizure, thoracic and cerebellar dysplasia	Strabismus, cutaneous plexiform schwannoma	Visual impairment, strabismus	Cutaneous plexiform schwannoma, wasting of the right hand muscles, cataract	Cataract, seizure	Strabismus	Cutaneous plexiform schwannoma
**Presenting feature/pathology**								
**Ophthalmological findings**								
Cataract	-	-	-	-	+	+	-	-
Retinal/Maculopathy	+	-	-	+	-	-	-	-
Strabismus	-	-	Idiopathic	Idiopathic	-	-	Idiopathic	-
Others	-	-	-	-	-	-	-	-
**Cutaneous features**								

CAL spots	-	+	+	-	-	-	-	-
Cutaneous plexiform schwannoma	+	+	+	-	+	-	-	+
**Cranial lesions**								
VS bilateral	-	-	-	-	-	-	-	-
VS unilateral	-	-	-	-	-	-	-	-
Cranial nerves	-	-	-	-	-	-	-	-
Meningioma	-	-	-	-	-	Temporal	-	-
Astrocytoma	-	-	-	-	-	-	-	-
Cortex dysplasia	-	Cerebellar	-	-	-	-	-	-
Vascular	-	-	-	-	-	-	-	-
Others	-	Bifrontal angiomatosis	-	-	-	-	-	-
**Spinal lesions**								
Ependymoma	-	-	-	-	-	-	-	-
Schwannoma	-	-	-	-	-	-	-	-
Meningioma	-	-	-	-	-	-	-	-
**Peripheral abnormalities**								
Peripheral nerve schwannoma	-	-	-	-	-	-	-	-
Neuropathy	-	-	-	-	+	-	-	-
**Others**	-	Thoracic dysplasia	-	-	-	-	-	-
**Incidental finding/asymptomatic**	-	-	-	-	-	-	-	-
**Symptom/Pathology leading to diagnosis (if different to presenting symptom)**	-	-	-	-	-	-	Hearing loss (VS)	Thoracic hypesthesia, back pain (spinal extramedullary tumors)

L = left, R = right, Age = median age in years, NA = not available, CAL = café-au-lait spots, BVZ = bevacizumab; MOS = mosaic, NO = no mutation, NS = nonsense mutation, FS = frameshift mutation, MIS = missense mutation, SP = splicing mutation, DEL = deletion, VS = vestibular schwannoma, SAH = subarachnoid hemorrhage.

	**Patient No**							
	**14**	**15**	**16**	**17**	**18**	**19**	**20**	**21**
**Sex**	M	F	F	F	F	F	M	M
**Family history**	Negative	Negative	Negative	Negative	Negative	Negative	Negative	Negative
***NF2* mutation type**	NS	SP	NS	NA	DEL	DEL	NA	SP
**Age at signs of symptoms**	1	6	11	11	5	16	11	0
**Age at time of diagnosis**	6	6	11	12	12	16	11	10
**Age at start of treatment (L/R)**	-/16	-/12	14/-	14/14	-/15	17/20	-/18	13/12

**Age at time of surgery (L/R)**	-/16	-/12	14/-	16/-	-/15	17/25	-/18	13/12
**Age at deafness (L/R)**	-/16	-/-	14/13	-/-	-/16	18/25	-/-	-/-
**BVZ in the following course**	No	No	No	Yes	No	Yes	No	No
**Radiation in the following course (L/R)**	No/No	No/No	No/No	No/No	No/No	No/No	No/No	No/No
**Presenting symptoms**	Cataract, epiretinal gliosis, paresis of the left arm	Strabismus, wasting of the right thigh muscles, cutaneous plexiform schwannoma	Facial palsy, hypacusis	Hypacusis	Peripheral nerve schwannoma, radiating pain	Back pain, gait disturbances, scoliosis	Cutaneous plexiform schwannoma, retinal hamartoma, cataract	Visual impairment
**Presenting feature/pathology**								
**Ophthalmological findings**								
Cataract	+	-	-	-	-	-	+	+
Retinal/Maculopathy	+	-	-	-	-	-	+	+
Strabismus	-	Tumor-associated	-	-	-	-	-	-
Others	-	-	-	-	-	-	-	-
**Cutaneous features**								
CAL spots	-	-	-	-	-	-	-	-
Cutaneous plexiform schwannoma	-	+	-	-	-	-	+	-
**Cranial lesions**								
VS bilateral	-	-	+	+	-	-	-	
VS unilateral	-	-	-	-	-	-	-	
Cranial nerves	-	III schwannoma	VII schwannoma	-	-	-	-	
Meningioma	-	craniocervical	-	-	-	-	-	
Cortex dysplasia	-	-	-	-	-	-	-	
Vascular	-	-	-	-	-	-	-	
Others	-	-	-	-	-	-	-	
**Spinal lesions**								
Ependymoma	-	-	-	-	-	+	-	-
Schwannoma	-	-	-	-	-	-	-	-
Meningioma	-	Craniocervical	-	-	-	-	-	-
**Peripheral abnormalities**								
Peripheral nerve schwannoma	-	-	-	-	+	-	-	-
Neuropathy	+	+	-	-	-	-	-	-
**Others**	-	-	-	-	-	Scoliosis	-	-
**Incidental finding/asymptomatic**	-	-	-	-	-	-	-	-

**Symptom/Pathology leading to diagnosis (if different to presenting symptom)**	Cutaneous plexiform schwannoma, cortex dysplasia	-	-	-	Hypacusis (VS)	-	-	-

	**Patient No**							
	**22**	**23**	**24**	**25**	**29**	**30**	**35**	**36**
**Sex**	F	F	M	M	F	F	F	M
**Family history**	Negative	Negative	Negative	Negative	Positive (mother)	Positive (father)	Negative	Negative
***NF2* mutation type**	NA	FS	SP	SP	MOS	NA	NA	FS
**Age at signs of symptoms**	14	5	1	1	17	14	13	17
**Age at time of diagnosis**	15	14	13	15	17	15	13	17
**Age at start of treatment (L/R)**	26/15	15/16	14/14	16/17	21/21	24/24	17/-	23/-
**Age at time of surgery (L/R)**	26/15	15/16	14/14	16/17	-/-	-/-	17/-	23/-
**Age at deafness (L/R)**	-/15	-/-	-/-	-/-	-/-	-/-	17/-	25/-
**BVZ in the following course**	No	Yes	No	No	Yes	Yes	No	No
**Radiation in the following course (L/R)**	No/No	No/No	No/No	No/No	No/No	No/No	No/No	No/No
**Presenting symptoms**	Strabismus, cutaneous plexiform schwannoma	Cutaneous plexiform schwannoma	Visual impairment, strabismus	Scoliosis	Scoliosis, cutaneous plexiform schwannoma, strabismus	Paresis of the right leg, cutaneous plexiform schwannoma	Cutaneous plexiform schwannoma, anisocoria	Hemihypesthesia, cognitive disorder, intracranial pressure symptoms
**Presenting feature/pathology**								
**Ophthalmological findings**								
Cataract	-	-	+	-	-	-	+	-
Retinal/Maculopathy	-	-	-	-	-	-	+	-
Strabismus	Idiopathic	-	Idiopathic	-	Idiopathic	-	-	-
Others	-	-	-	-	-	-	Idiopathic anisocoria	-
**Cutaneous features**								
CAL spots	-	-	-	-	-	-	-	-
Cutaneous plexiform schwannoma	+	+	-	-	+	+	+	-
**Cranial lesions**								

VS bilateral	-	-	-	-	-	-	-	-
VS unilateral	-	-	-	-	-	-	-	-
Cranial nerves	-	-	-	-	-	-	-	-
Meningioma	-	-	-	-	-	-	-	-
Cortex dysplasia	-	-	-	-	-	-	-	-
Vascular	-	-	-	-	-	-	-	SAH, pericallosal artery aneurysm
Others	-	-	-	-	-	-	-	-
**Spinal lesions**								
Ependymoma	-	-	-	-	-	-	-	-
Schwannoma	-	-	-	-	-	-	-	-
Meningioma	-	-	-	-	-	-	-	-
**Peripheral abnormalities**								
Peripheral nerve schwannoma	-	-	-	-	-	+	-	-
Neuropathy	-	-	-	-	-	-	-	-
**Others**	-	-	-	Scoliosis	Scoliosis	-	-	-
**Incidental finding/asymptomatic**	-	-	-	-	-	-	-	-
**Symptom/Pathology leading to diagnosis (if different to presenting symptom)**	-	Dizziness, tinnitus (VS)	Dizziness, gait and taste disturbances, ataxia, dysarthria, facial palsy, hypacusis ischemic stroke left ponto-medullary transition and VS)	Dizziness, hypacusis (VS)	Positive family history	Positive family history	-	-

	**Patient No**							
	**37**	**38**	**40**	**42**	**45**	**48**	**49**	**51**
**Sex**	M	M	F	F	M	F	M	M
**Family history**	Negative	Negative	Positive (father)	Negative	Negative	Negative	Negative	Negative
**NF 2 mutation type**	FS	NA	NO	NS	SP	SP	DEL	MOS
**Age at signs of symptoms**	18	5	11	Asymptomatic	7	1	1	17
**Age at time of diagnosis**	18	6	11	10	7	1	1	17
**Age at start of treatment (L/R)**	19/23	16 and 25/25	-/-	-/-	21/20	16/-	-/-	30/-
**Age at time of surgery (L/R)**	19/23	16 and 25/25	-/-	-/-	21/20	16/-	-/-	30/-
**Age at deafness (L/R)**	19/23	16/-	-/-	-/-	-/-	-/-	-/-	-/-

**BVZ in the following course**	Yes	Yes	No	No	No	No	No	No
**Radiation in the following course (L/R)**	No/No	No/No	No/No	No/No	No/No	No/No	No/No	No/No
**Presenting symptoms**	Facial palsy	Facial palsy, hypacusis, cavus foot, cutaneous plexiform schwannoma, amblyopia, epiretinal gliosis	Asymptomatic, positive family history	Asymptomatic, incidental finding (post-trauma)	Hemiparesis, dysarthria	Strabismus, papilledema	Visual impairment	Strabismus, visual loss, facial palsy
**Presenting feature/pathology**								
**Ophthalmological findings**								
Cataract	-	-	-	-	-	-	+	-
Retinal/Maculopathy	-	+	-	-	-	-	-	-
Strabismus	-	-	-	-	-	-	-	Idiopathic
Others	-	Amblyopia	-	-	-	Idiopathic papilledema	-	-
**Cutaneous features**								
CAL spots	-	-	-	-	-	-	-	-
Cutaneous plexiform schwannoma	-	+	-	-	-	-	-	-
**Cranial lesions**								
VS bilateral	+	+	-	-	-	-	-	-
VS unilateral	-	-	-	-	-	-	-	-
Cranial nerves	-	-	-	-	-	-	-	-
Meningioma	-	-	-	-	-	Frontal	-	-
Cortex dysplasia	-	-	-	-	-	-	-	-
Vascular	-	-	-	-	Ischemic stroke pons right	-	-	-
Others	-	-	-	-	-	-	-	-
**Spinal lesions**								
Ependymoma	-	-	-	-	-	-	-	-
Schwannoma	-	-	-	-	-	-	-	-
Meningioma	-	-	-	-	-	-	-	-
**Peripheral abnormalities**								
Peripheral nerve schwannoma	-	-	-	-	-	-	-	-
Neuropathy	-	-	-	-	-	-	-	-
**Others**	-	Cavus foot	-	-	--	-	-	-

**Incidental finding/asymptomatic**	-	-	-	Post trauma	-	-	-	-
**Symptom/Pathology leading to diagnosis (if different to presenting symptom)**	-	-	Positive family history	Post trauma (spinal tumors)	-	-	-	-

	**Patient No**							
	**53**	**60**	**62**	**63**	**67**	**68**	**94**	**106**
**Sex**	M	M	F	F	F	M	M	F
**Family history**	Negative	Negative	Negative	Negative	Negative	Negative	Positive (father)	Negative
**NF 2 mutation type**	NA	NA	SP	NA	NA	NA	SP	NA
**Age at signs of symptoms**	8	Asymptomatic	14	10	2	4	8	15
**Age at time of diagnosis**	12	16	15	15	9	7	8	18
**Age at start of treatment (L/R)**	-/13	20,	15/16	16/15	28/28	-/-	25/13	20/22
**Age at time of surgery (L/R)**	-/13	20 and 23/16 and 18	15/-	16/15	28/28	-/-	25/13	20/22
**Age at deafness (L/R)**	-/-	23/16	16/-	-/31	-/17	-/-	-/-	20/-
**BVZ in the following course**	No	No	No	No	No	No	No	No
**Radiation in the following course (L/R)**	No/No	No/No	No/No	No/No	No/No	No/No	No/No	No/No
**Presenting symptoms**	Facial palsy	Incidental finding	Cutaneous plexiform schwannoma, strabismus, peripheral nerve schwannoma	Cutaneous plexiform schwannoma, positive family history	Cutaneous plexiform schwannoma, visual loss, amblyopia left	Cutaneous plexiform schwannoma, proptosis, visual loss	Cutaneous plexiform schwannoma, cataract	Hypacusis, tinnitus, dizziness, gait disturbances
**Presenting feature/pathology**								
**Ophthalmological findings**								
Cataract	-	-	-	-	-	-	+	-
Retinal/Maculopathy	-	-	-	-	-	-	-	-
Strabismus	-	-	Tumor-associated	-	-	-	-	-
Others	-	-	-	-	Amblyopia	-	-	-
**Cutaneous features**								
CAL spots	-	-	-	-	-	-	-	-

Cutaneous plexiform schwannoma	-	-	+	+	+	+	+	-
**Cranial lesions**								
VS lesions	+	-	-	-	-	-	-	+
VS unilateral	-	-	-	-	-	-	-	-
Cranial nerves	-	-	III schwannoma	-	-	-	-	-
Meningioma	-	-	-	-	-	Intraorbital	-	-
Cortex dysplasia	-	-	-	-	-	-	-	-
Vascular	-	-	-	-	-	-	-	-
Other	-	-	-	-	-	-	-	-
**Spinal lesions**								
Ependymoma	-	-	-	-	-	-	-	-
Schwannoma	-	-	-	-	-	-	-	-
Meningioma	-	-	-	-	-	-	-	-
**Peripheral abnormalities**								
Peripheral nerve schwannoma	-	-	+	-	-	-	-	-
Neuropathy	-	-	-	-	-	-	-	-
**Others**	-	-	-	-	-	-	-	-
**Incidental finding/asymptomatic**	-	+	-	-	-	-	-	-
**Symptom/Pathology leading to diagnosis (if different to presenting symptom)**	-	-	Hypacusis, gait disturbances, tinnitus (VS)	Positive family history	-	-	-	-

	**Patient No**							
	**109**	**111**	**117**	**120**	**122**	**129**	**130**	**136**
**Sex**	M	M	M	F	M	F	F	M
**Family history**	Negative	Negative	Negative	Positive (mother, aunt, sister, cousin)	Positive (father)	Negative	Negative	Negative
***NF2* mutation type**	NA	NA	DEL	FS	FS	FS	No	NA
**Age at signs of symptoms**	16	17	15	14	10	10	7	16
**Age at time of diagnosis**	16	17	15	14	10	13	7	16
**Age at start of treatment (L/R)**	-/17	19/19	26/15	27/22 and 28	-/-	15/14	14/-	25/16
**Age at time of surgery (L/R)**	-/17	19/19	27/-	27/22 and 28	-/-	15/14	14/-	25/16
**Age at deafness (L/R)**	Unknown/unknown	-/23	-/16	-/-	-/-	-/-	-/-	-/-
**BVZ in the following course**	No	No	Yes	No	No	No	No	No

**Radiation in the following course (L/R)**	No/No	No/No	No/No	No/No	No/No	No/No	No/No	No/No
**Presenting symptoms**	Hypacusis	Hypacusis, hoarseness	Cutaneous plexiform schwannoma, strabismus, CAL	Hypacusis	Hypacusis	Cutaneous plexiform schwannoma, hypacusis	Paresis left arm	Hypacusis
**Presenting feature/pathology**								
**Ophthalmological findings**								
Cataract	-	-	-	-	-	-	-	-
Retinal/Maculopathy	-	-	-	-	-	-	-	-
Strabismus	-	-	Idiopathic	-	-	-	-	-
Others	-	-	-	-	-	-	-	-
**Cutaneous features**								
CAL spots	-	-	+	-	-	-	-	-
Cutaneous plexiform schwannoma	-	-	+	-	-	-	-	-
**Cranial lesions**								
VS bilateral	+	+	-	+	+	+	-	+
VS unilateral	-	-	-	-	-	-	-	-
Cranial nerves	-	-	-	-	-	-	-	-
Meningioma	-	-	-	-	-	-	-	-
Cortex dysplasia	-	-	-	-	-	-	-	-
Vascular	-	-	-	-	-	-	-	-
Others	-	-	-	-	-	-	-	-
**Spinal lesions**								
Ependymoma	-	-	-	-	-	-	-	-
Schwannoma	-	-	-	-	-	-	-	-
Meningioma	-	-	-	-	-	-	-	-
**Peripheral abnormalities**								
Peripheral nerve schwannoma	-	-	-	-	-	-	+	-
Neuropathy	-	-	-	-	-	-	-	-
**Others**	-	-	-	-	-	-	-	-
**Incidental finding/asymptomatic**	-	-	-	-	-	-	-	-
**Symptom/Pathology leading to diagnosis (if different to presenting symptom)**	-	-	Facial palsy (VS)	-	-	-	-	-

	**Patient No**							
	**137**	**141**	**144**	**152**	**156**	**159**	**162**	**166**
**Sex**	F	F	M	M	M	F	F	M
**Family history**	Negative	Negative	Positive (mother)	Positive (brother)	Negative	Negative	Negative	Positive (mother)
***NF2* mutation type**	NA	NA	NA	FS	DEL	FS	NS	FS

**Age at signs of symptoms**	4	3	4	11	13	17	14	0
**Age at time of diagnosis**	11	13	6	11	18	17	14	11
**Age at start of treatment (L/R)**	23/11	16/16	-/-	-/-	29/19	-/-	18/18	14/15
**Age at time of surgery (L/R)**	23/11	16/16	-/-	-/-	29/19	-/-	23/23	14/15
**Age at deafness (L/R)**	-/22	-/-	Unknown/ unknown	-/-	Unknown/ unknown	-/-	-/-	-/-
**BVZ in the following course**	No	No	No	No	Yes	No	Yes	Yes
**Radiation in the following course (L/R)**	No/No	No/No	No/No	No/No	Yes/No	No/No	No/No	No/No
**Presenting symptoms**	Strabismus	Cutaneous plexiform schwannoma, seizure	Cutaneous plexiform schwannoma, radiating pain	Facial palsy, strabismus, positive family history	Back pain	Proptosis	Cutaneous plexiform schwannoma, proptosis	Cutaneous plexiform schwannoma, strabismus, visual loss
**Presenting feature/pathology**								
**Ophthalmological findings**								
Cataract	-	-	-	-	-	-	-	-
Retinal/Maculopathy	-	-	-	-	-	-	-	+
Strabismus	Idiopathic	-	-	Tumor-associated	-	-	-	Idiopathic
Others	-	-	-	-	-	-	-	-
**Cutaneous features**								
CAL spots	-	-	-	-	-	-	-	-
Cutaneous plexiform schwannoma	-	+	+	-	-	-	+	+
**Cranial lesions**								
VS bilateral	-	+	-	-	-	-	-	-
VS unilateral	-	-	-	-	-	-	-	-
Cranial nerves	-	-	-	VII and III schwannomas	-	Periorbital schwannoma	Periorbital Achwannoma	-
Meningioma	-	Temporal	-	-	-	-	-	-
Cortex dysplasia	-	-	-	-	-	-	-	-
Vascular	-	-	-	-	-	-	-	-
Others	-	-	-	-	-	-	-	-
**Spinal lesions**								
Ependymoma	-	-	-	-	-	-	-	-
Schwannoma	-	-	-	-	+	-	-	-
Meningioma	-	-	-	-	-	-	-	-
**Peripheral abnormalities**								
Peripheral nerve schwannoma	-	-	+	-	-	-	-	-
Neuropathy	-	-	-	-	-	-	-	-
**Others**	-	-	-	-	-	-	-	-

**Incidental finding/asymptomatic**	-	-	-	-	-	-	-	-
**Symptom/Pathology leading to diagnosis (if different to presenting symptom)**	Hypacusis, dizziness (VS)	-	-	-	-	-	-	-

	**Patient No**				
	**167**	**168**	**172**	**178**	**179**
**Sex**	M	M	M	F	M
**Family history**	Negative	Positive (father)	Negative	Negative	Negative
***NF2* mutation type**	NA	FS	FS	No	NS
**Age at signs of symptoms**	16	16	11	9	0
**Age at time of diagnosis**	16	16	11	9	5
**Age at start of treatment (L/R)**	-/-	-/29	15/15	-/-	-/-
**Age at time of surgery (L/R)**	-/-	-/29	19/20	-/-	-/-
**Age at deafness (L/R)**	-/-	-/-	-/-	-/-	-/-
**BVZ in the following course**	No	No	Yes	No	No
**Radiation in the following course (L/R)**	No/No	No/No	No/No	No/No	No/No
**Presenting symptoms**	Hypesthesia right leg	Cataract	Visual loss	Foot lift plegia right	Strabismus, visual loss
**Presenting feature/pathology**					
**Ophthalmological findings**					
Cataract	-	+	-	-	-
Retinal/Maculopathy	-	-	-	-	-
Strabismus	-	-	-	-	Idiopathic
Others	-	-	-	-	-
**Cutaneous features**					
CAL spots	-	-	-	-	-
Cultaneous plexiform schwannoma	-	-	-	-	-
**Cranial lesions**					
VS bilateral	-	-		-	-
VS unilateral	-	-	-	-	-
Cranial nerves	-	-	-	-	-
Meningioma	-	-	Optic nerve sheath	-	Optic nerve sheath
Cortex dysplasia	-	-	-	-	-
Vascular	-	-	-	-	-
Others	-	-	-	-	-
**Spinal lesions**					

Ependymoma	-	-	-	-	-
Schwannoma	-	-	-	-	-
Meningioma	Thoracic	-	-	-	-
**Peripheral abnormalities**					
Peripheral nerve schwannoma	-	-	-	-	-
Neuropathy	-	-	-	+	-
**Others**	-	-	-	-	-
**Incidental finding/asymptomatic**	-	-	-	-	-
**Symptom/Pathology leading to diagnosis (if different to presenting symptom)**	-	-	-	-	-

	**Patient No**							
	**10**	**12**	**13**	**26**	**27**	**28**	**31**	**33**
**Sex**	F	F	M	F	F	F	F	F
**Family history**	Negative	Negative	Negative	Negative	Negative	Positive (grandfather)	Positive (father)	Negative
***NF2* mutation type**	No	SP	MOS	SP	SP	MOS	NA	MOS
**Age at signs of symptoms**	21	22	14	20	31	22	36	10
**Age at time of diagnosis**	22	22	26	21	36	22	36	29
**Age at start of treatment (L/R)**	22/23	24/23	30/30	-/26	-/36	-/23	58/58	31/31
**Age at time of surgery (L/R)**	22/23	24/23	30/30	-/26	-/36	-/23	-/-	34/-
**Age at deafness (L/R)**	-/25	-/-	-/-	-/-	-/39	27/26	-/-	-/-
**BVZ in the following course**	Yes	Yes	Yes	no	Yes	No	Yes	Yes
**Radiation in the following course (L/R)**	No/No	No/No	No/No	No/No	No/No	No/No	No/No	No/No
**Presenting symptoms**	Cutaneous plexiform schwannoma, CAL	Hemiparesis, dizziness, facial palsy, dysarthria, fine motor skill disorders	Wasting of the left hand muscles, cutaneous plexiform schwannoma	Seizure	Dizziness, sudden hearing loss	Headache, dizziness	Asymptomatic	Cutaneous plexiform schwannoma
**Presenting feature/pathology**								
**Ophthalmological findings**								
Cataract	-	-	-	-	-	-	-	-
Retinal/Maculopathy	-	-	-	-	-	-	-	-
Strabismus	-	-	-	-	-	-	-	-
Others	-	-	-	-	-	-	-	-
**Cutaneous features**								
CAL spots	+	-	-	-	-	-	-	-
Cultaneous plexiform schwannoma	+	-	+	-	-	-	-	+
**Cranial lesions**								

VS bilateral	-	-	-	-	+	-	-	-
VS unilateral	-	-	-	-	-	-	-	-
Cranial nerves	-	-	-	-	-	-	-	-
Meningioma	-	-	-	Frontal	-	Confluens sinuum	-	-
Cortex dysplasia	-	-	-	-	-	-	-	-
Vascular	-	Ischemic stroke left cerebellar peduncle	-	-	-	-	-	-
Others	-	-	-	-	-	-	-	-
**Spinal lesions**								
Ependymoma	-	-	-	-	-	-	-	-
Schwannoma	-	-	-	-	-	-	-	-
Meningioma	-	-	-	-	-	-	-	-
**Peripheral abnormalities**								
Peripheral nerve schwannoma	-	-	-	-	-	-	-	-
Neuropathy	-	-	-	-	-	-	-	-
**Others**	-	-	-	-	-	-	-	-
**Incidental finding/asymptomatic**	-	-	-	-	-	-	-	-
**Symptom/Pathology leading to diagnosis (if different to presenting symptom)**	Hypacusis, tinnitus (VS)	-	Tinnitus, balance disorders (VS)	-	-	-	Positive family history	Cauda equina syndrome (ependymoma), tinnitus (VS)

	**Patient No**							
	**34**	**39**	**41**	**43**	**44**	**56**	**64**	**66**
**Sex**	M	F	M	F	F	M	F	F
**Family history**	Negative	Positive (father)	Positive (daughter)	Negative	Negative	Negative	Negative	Negative
***NF2* mutation type**	NA	NS	MOS	MIS	MOS	MOS	NA	NA
**Age at signs of symptoms**	31	20	27	32	20	24	NA	23
**Age at time of diagnosis**	31	20	29	32	20	28	34	44
**Age at start of treatment (L/R)**	-/40	23/23	34/30	33/43	40/-	32/-	-/-	44/32
**Age at time of surgery (L/R)**	-/40	23/23	34/30	33/-	-/-	32/-	-/-	44/32
**Age at deafness (L/R)**	-/-	24/-	-/-	-/-	-/-	-/-	41/-	-/-
**BVZ in the following course**	No	Yes	No	Yes	No	No	No	No
**Radiation in the following course (L/R)**	No/No	No/No	No/No	No/No	Yes/No	No/No	No/No	No/No

**Presenting symptoms**	Hypacusis, sudden hearing loss, dizziness	Peripheral nerve schwannoma, hypacusis	Sudden hearing loss, hypacusis, tinnitus	Sudden hearing loss, hypacusis, tinnitus	Multiple subcutaneous peripheral nerve schwannomas lower extremity	Multiple subcutaneous peripheral nerve schwannomas	Incidental finding (unspecific neck pain)	Incidental finding
**Presenting feature/pathology**								
**Ophthalmological findings**								
Cataract	-	-	-	-	-	-	-	-
Retinal/Maculopathy	-	-	-	-	-	-	-	-
Strabismus	-	-	-	-	-	-	-	-
Others	-	-	-	-	-	-	-	-
**Cutaneous features**								
CAL spots	-	-	-	-	-	-	-	-
Cultaneous plexiform schwannoma	-	-	-	-	-	-	-	-
**Cranial lesions**								
VS bilateral	+	+	+	+	-	-	-	-
VS unilateral	-	-	-	-	-	-	-	-
Cranial nerves	-	-	-	-	-	-	-	-
Meningioma	-	-	-	-	-	-	-	-
Cortex dysplasia	-	-	-	-	-	-	-	-
Vascular	-	-	-	-	-	-	-	-
Others	-	-	-	-	-	-	-	-
**Spinal lesions**								
Ependymoma	-	-	-	-	-	-	-	-
Schwannoma	-	-	-	-	-	-	-	-
Meningioma	-	-	-	-	-	-	-	-
**Peripheral abnormalities**								
Peripheral nerve schwannoma	-	+	-	-	+	+	-	-
Neuropathy	-	-	-	-	-	-	-	-
**Others**	-	-	-	-	-	-	-	-
**Incidental finding/asymptomatic**	-	-	-	-	-	-	+	+
**Symptom/Pathology leading to diagnosis (if different to presenting symptom)**	-	-	-	-	-	-	-	-

	**Patient No**							
	**70**	**79**	**86**	**87**	**88**	**95**	**97**	**107**
**Sex**	F	M	F	F	M	M	F	M

**Family history**	Negative	Positive (mother, sister)	Negative	Negative	Negative	Negative	Positive (daughter)	Negative
***NF*2 mutation type**	NA	MOS	NA	NA	NA	NA	NA	MOS
**Age at signs of symptoms**	1	18	22	21	28	14	46	29
**Age at time of diagnosis**	39	21	24	22	32	20	25	29
**Age at start of treatment (L/R)**	-/-	Unknown/40	29/-	23/-	32/33	39/21	47/NA	-/40
**Age at time of surgery (L/R)**	-/-	-/40	29/-	23/-	32/33	39/21	47/NA	-/40
**Age at deafness (L/R)**	-/-	Unknown/34	-/-	23/unknown	-/-	-/30	-/-	Unknown/-
**BVZ in the following course**	No	No	No	No	Yes	No	No	No
**Radiation in the following course (L/R)**	No/No	Yes/No	No/No	No/No	No/No	No/No	No/No	No/No
**Presenting symptoms**	Cutaneous plexiform schwannoma, multiple subcutaneous peripheral nerve schwannoma	Headache, wasting and weakness of the left arm	Cutaneous plexiform schwannoma	Headache, incidental finding	Headache, hypacusis	Incidental finding (family history for seizures)	Breathlessness	Tinnitus, hypacusis, CAL
**Presenting feature/pathology**								
**Ophthalmological findings**								
Cataract	-	-	-	-	-	-	-	-
Retinal/Maculopathy	-	-	-	-	-	-	-	-
Strabismus	-	-	-	-	-	-	-	-
Others	-	-	-	-	-	-	-	-
**Cutaneous features**								
CAL spots	-	-	-	-	-	-	-	+
Cultaneous plexiform schwannoma	+	-	+	-	-	-	-	-
**Cranial lesions**								
VS bilateral	-	-	-	-	+	-	-	+
VS unilateral	-	-	-	-	-	-	-	-
Cranial nerves	-	-	-	-	-	-	-	-
Meningioma	-	-	-	-	-	-	-	-
Cortex dysplasia	-	-	-	-	-	-	-	-
Vascular	-	-	-	-	-	-	-	-
Others	-	-	-	-	-	-	-	-
**Spinal lesions**								
Ependymoma	-	+	-	-	-	-	-	-
Schwannoma	-	-	-	-	-	-	-	-
Meningioma	-	-	-	-	-	-	-	-
**Peripheral abnormalities**								
Peripheral nerve schwannoma	+	-	-	-	-	-	+	-
Neuropathy	-	-	-	-	-	-	-	-
**Others**	-	-	-	-	-	-	-	-

**Incidental finding/asymptomatic**	-	-	-	+	-	+	-	-
**Symptom/Pathology leading to diagnosis (if different to presenting symptom)**	-	CAL, multiple subcutaneous peripheral nerve schwannoma	Hypacusis, tinnitus, gait disturbances (VS)	-	-	-	-	-

	Patient No							
	**108**	**110**	**113**	**114**	**115**	**116**	**121**	**132**
**Sex**	M	M	M	M	F	M	F	M
**Family history**	Negative	Negative	Negative	Positive (father)	Negative	Negative	Negative	Positive (mother)
***NF2* mutation type**	MOS	NA	NA	MIS	NA	NA	NA	MOS
**Age at signs of symptoms**	22	23	19	37	19	19	15	19
**Age at time of diagnosis**	29	23	22	37	19	23	36	19
**Age at start of treatment (L/R)**	31/ -	24/-	22/28	46/46	27/32	28 and 39/32	36/-	24/23
**Age at time of surgery (L/R)**	31/ -	24/-	22/28	46/46	27/32	28 and 39/32	36/-	24/23
**Age at deafness (L/R)**	unknown, unknown	24/unknown	22/-	-/-	27/32	Unknown/ unknown	56/-	Unknown/24
**BVZ in the following course**	No	No	No	No	No	No	No	No
**Radiation in the following course (L/R)**	No/No	No/No	No/No	No/No	No/Yes	No/No	No/No	No/No
**Presenting symptoms**	Seizure, papilledema, cutaneous plexiform schwannoma	Hypacusis	Hypacusis, cutaneous plexiform schwannoma	Hypacusis, tinnitus, gait disturbances, facial palsy	Hypacusis, intracranial pressure symptoms	Hypacusis	Sudden hearing loss, Facial palsy	Hypacusis, tinnitus
**Presenting feature/pathology**								
**Ophthalmological findings**								
Cataract	-	-	-	-	-	-	-	-
Retinal/Maculopathy	-	-	-	-	-	-	-	-
Strabismus	-	-	-	-	-	-	-	-
Others	Idiopathic papilledema	-	-	-	-	-	-	-
**Cutaneous features**								
CAL spots	-	-	-	-	-	-	-	-
Cultaneous plexiform schwannoma	+	-	+	-	-	-	-	-
**Cranial lesions**								
VS bilateral	-	+	+	+	+	+	+	+
VS unilateral	-	-	-	-	-	-	-	-

Cranial nerves	-	-	-	VII schwannoma	-	-	-	-
Meningioma	-	-	-	-	-	-	-	-
Cortex dysplasia	-	-	-	-	-	-	-	-
Vascular	-	-	-	-	-	-	-	-
Others	-	-	-	-	-	-	-	-
**Spinal lesions**								
Ependymoma	-	-	-	-	-	-	-	-
Schwannoma	-	-	-	-	-	-	-	-
Meningioma	-	-	-	-	-	-	-	-
**Peripheral abnormalities**								
Peripheral nerve schwannoma	-	-	-	-	-	-	-	-
Neuropathy	-	-	-	-	-	-	-	-
**Others**	-	-	-	-	-	-	-	-
**Incidental finding/asymptomatic**	-	-	-	-	-	-	-	-
**Symptom/Pathology leading to diagnosis (if different to presenting symptom)**	-	-	-	-	-	-	-	-

	**Patient No**							
	**138**	**140**	**147**	**148**	**153**	**154**	**157**	**161**
**Sex**	M	M	F	M	F	M	F	M
**Family history**	Negative	Negative	Negative	Positive (mother)	Negative	Negative	Negative	Negative
***NF2* mutation type**	NA	NA	MOS	NA	NS	NA	MOS	No
**Age at signs of symptoms**	39	18	41	27	26	31	32	22
**Age at time of diagnosis**	39	27	41	27	26	31	32	22
**Age at start of treatment (L/R)**	47/45	31/27	-/42	46/46	26/27	-/19	-/32	-/-
**Age at time of surgery (L/R)**	47/45	31/27	-/42	46/46	26/27	-/40	-/32	-/-
**Age at deafness (L/R)**	48/48	32/30	42/35	-/-	22/27	-/-	-/-	-/-
**BVZ in the following course**	No	Yes	No	No	Yes	No	No	No
**Radiation in the following course (L/R)**	No/No	No/No	No/No	No/No	No/No	No/Yes	No/No	No/No
**Presenting symptoms**	Tinnitus	Visual impairment	Tinnitus	Hypacusis, balance disorders, headache, double vision	Hypacusis, visual loss,	Sudden hearing loss	Hypacusis	Strabismus
**Presenting feature/pathology**								
**Ophthalmological findings**								
Cataract	-	+	-	-	-	-	-	-
Retinal/Maculopathy	-	-	-	-	-	-	-	-
Strabismus	-	-	-	-	-	-	-	Tumor-associated
Others	-	-	-	-	-	-	-	-
**Cutaneous features**								

CAL spots	-	-	-	-	-	-	-	-
Cultaneous plexiform schwannoma	-	-	-	-	-	-	-	-
**Cranial lesions**								
VS bilateral	+	-	+	+	+	+	+	-
VS unilateral	-	-	-	-	-	-	-	-
Cranial nerves	-	-	-	-	-	-	-	-
Meningioma	-	-	-	-	Optic nerve sheath	-	-	Temporal horn left lateral ventricle
Cortex dysplasia	-	-	-	-	-	-	-	-
Vascular	-	-	-	-	-	-	-	-
Others	-	-	-	-	-	-	-	-
**Spinal lesions**								
Ependymoma	-	-	-	-	-	-	-	-
Schwannoma	-	-	-	-	-	-	-	-
Meningioma	-	-	-	-	-	-	-	-
**Peripheral abnormalities**								
Peripheral nerve schwannoma	-	-	-	-	-	-	-	-
Neuropathy	-	-	-	-	-	-	-	-
**Others**	-	-	-	-	-	-	-	-
**Incidental finding/asymptomatic**	-	-	-	-	-	-	-	-
**Symptom/Pathology leading to diagnosis (if different to presenting symptom)**	-	Hypacusis, dizziness (VS)-	-	-	-	-	-	-

	Patient No				
	**164**	**169**	**170**	**174**	**175**
**Sex**	M	F	M	F	M
**Family history**	Positive (daughter)	Negative	Negative	Negative	Negative
***NF2* mutation type**	NA	No	NA	NA	No
**Age at signs of symptoms**	35	24	21	35	39
**Age at time of diagnosis**	35	26	21	35	39
**Age at start of treatment (L/R)**	35/-	-/27	24/-	56/-	-/-
**Age at time of surgery (L/R)**	35/-	-/27	24/-	56/-	-/-
**Age at deafness (L/R)**	-/-	-/-	-/-	57/59	-/-
**BVZ in the following course**	no	no	no	no	no
**Radiation in the following course (L/R)**	no	no	no	no	no
**Presenting symptoms**	Hypacusis	Hypacusis, facial palsy	Tinnitus	Tinnitus	Radiating pain (C7)
**Presenting feature/pathology**					
**Ophthalmological findings**					
Cataract	-	-	-	-	-

Retinal/Maculopathy	-	-	-	-	-
Strabismus	-	-	-	-	-
Others	-	-	-	-	-
**Cutaneous features**					
CAL spots	-	-	-	-	-
Cultaneous plexiform schwannoma	-	-	-	-	-
**Cranial lesions**					
VS bilateral	+	+	+	+	-
VS unilateral	-	-	-	-	-
Cranial nerves	-	VII schwannoma	-	-	-
Meningioma	-	-	-	-	-
Cortex dysplasia	-	-	-	-	-
Vascular	-	-	-	-	-
Others	-	-	-	-	-
**Spinal lesions**					
Ependymoma	-	-	-	-	-
Schwannoma	-	-	-	-	-
Meningioma	-	-	-	-	-
**Peripheral abnormalities**					
Peripheral nerve schwannoma	-	-	-	-	+
Neuropathy	-	-	-	-	-
**Others**	-	-	-	-	-
**Incidental finding/asymptomatic**	-	-	-	-	-
**Symptom/Pathology leading to diagnosis (if different to presenting symptom)**	-	-	-	-	-

© 2020 by the authors. Licensee MDPI, Basel, Switzerland. This article is an open access article distributed under the terms and conditions of the Creative Commons Attribution (CC BY) license (http://creativecommons.org/licenses/by/4.0/).
